# Effectiveness of intensive educational sessions for nursing staff in large ICU on multi drug resistant organism’s acquisition rate: 3 year prospective observational study

**DOI:** 10.1186/1753-6561-5-S6-P140

**Published:** 2011-06-29

**Authors:** AS Alhenn, Ali Albraak, Amin Yousef, Mohammad Mhawesh, Noor Alqudah

**Affiliations:** 1 Previntion and control department, KING SAUD MEDICAL CITY, Riyadh, Saudi Arabia

## Introduction / objectives

***Introduction:*** unit-targeted, and informal educational interventions were included in several studies. The focus of the interventions was to encourage the behaviors change through improved understanding of the problem of multi drug resistant organisms (MDRO). Whether the desire change involved hand hygiene, or other outcomes, enhancing understanding and creating a culture that supported and promoted the desired behaviors, were viewed as essential to the success of the intervention.

***Setting:***A 100 Bed capacity Intensive care unit (ICU) in King Saud medical city in Riyadh.

## Methods

A 3-year, unit-targeted, prospective observational study was conducted to assess impact of intensive educational activities -for nursing staff in ICU- on MDRO’s rate. In-service educational session have been distributed to three cycles per year, each cycle contain (10) deferent topics about infection control policy in hospital in order of topic/week. Each topic was presented to (5-8) nursing staff in bed side area one time per day in each ICU section for about (10-15 min.). Same topics are presented in each cycle in order to insure maximum staff attendance from ICU staff at the end of the year.

## Result

Total of (1720) educational session have been presented betweenÂ (5/2008-12/2010) for (370) nurse per year annually. Attendance rate for were 78% and MDRO’s rate was decreased from 51.8 per 1000 to 25.3 per 1000 patients’ day, with attributable risk 48.8 %.

## Conclusion

***Conclusion:*** Provide education and training on risks and prevention of MDRO transmission during periodic educational updates, include information on organizational experience with MDROs and preventions strategies, is ensuring that systems are in place to promote optimal treatment of infections and MDROs acquisition rate.

## Disclosure of interest

None declared.

